# Can the SARS-CoV-2 Omicron Variant Confer Natural Immunity against COVID-19?

**DOI:** 10.3390/molecules27072221

**Published:** 2022-03-29

**Authors:** Abdul Hawil Abas, Siti Marfuah, Rinaldi Idroes, Diah Kusumawaty, Fatimawali, Moon Nyeo Park, Abolghasem Siyadatpanah, Fahad A. Alhumaydhi, Shafi Mahmud, Trina Ekawati Tallei, Talha Bin Emran, Bonglee Kim

**Affiliations:** 1 Department of Biology, Faculty of Mathematics and Natural Sciences, Sam Ratulangi University, Manado 95115, North Sulawesi, Indonesia; abdulabas102@student.unsrat.ac.id (A.H.A.); stmrfuah02@gmail.com (S.M.); 2 Department of Pharmacy, Faculty of Mathematics and Natural Sciences, Universitas Syiah Kuala, Kopelma Darussalam, Banda Aceh 23111, Aceh, Indonesia; rinaldi.idroes@unsyiah.ac.id; 3 Department of Biology, Faculty of Mathematics and Natural Sciences Education, Universitas Pendidikan Indonesia, Bandung 40154, West Java, Indonesia; diah.kusumawaty@upi.edu; 4 Pharmacy Study Program, Faculty of Mathematics and Natural Sciences, Sam Ratulangi University, Manado 95115, North Sulawesi, Indonesia; fatimawali@unsrat.ac.id; 5 College of Korean Medicine, Kyung Hee University, Hoegidong Dongdaemungu, Seoul 05253, Korea; mnpark@khu.ac.kr; 6 Ferdows School of Paramedical and Health, Birjand University of Medical Sciences, Birjand 97178-53577, Iran; asiyadatpanah@yahoo.com; 7 Department of Medical Laboratories, College of Applied Medical Sciences, Qassim University, Buraydah 52571, Saudi Arabia; f.alhumaydhi@qu.edu.sa; 8 Department of Genome Science, John Curtin School of Medical Research, Australian National University, Canberra, ACT 0200, Australia; shafimahmudfz@gmail.com; 9 Department of Pharmacy, BGC Trust University Bangladesh, Chittagong 4381, Bangladesh

**Keywords:** COVID-19, SARS-CoV-2, Omicron, natural immunity, hybrid immunity

## Abstract

The coronavirus disease 2019 (COVID-19) pandemic is still ongoing, with no signs of abatement in sight. The severe acute respiratory syndrome coronavirus 2 (SARS-CoV-2), which is the causative agent of this pandemic and has claimed over 5 million lives, is still mutating, resulting in numerous variants. One of the newest variants is Omicron, which shows an increase in its transmissibility, but also reportedly reduces hospitalization rates and shows milder symptoms, such as in those who have been vaccinated. As a result, many believe that Omicron provides a natural vaccination, which is the first step toward ending the COVID-19 pandemic. Based on published research and scientific evidence, we review and discuss how the end of this pandemic is predicted to occur as a result of Omicron variants being surpassed in the community. In light of the findings of our research, we believe that it is most likely true that the Omicron variant is a natural way of vaccinating the masses and slowing the spread of this deadly pandemic. While the mutation that causes the Omicron variant is encouraging, subsequent mutations do not guarantee that the disease it causes will be less severe. As the virus continues to evolve, humans must constantly adapt by increasing their immunity through vaccination.

## 1. Introduction

At the moment, any discussion of viruses will include severe acute respiratory syndrome coronavirus 2 (SARS-CoV-2), the virus that caused the global COVID-19 pandemic. As of19:50 p.m. CET on 11 March 2022, the WHO had confirmed 452,052,304 cases of COVID-19 worldwide, including 6,027,059 deaths. As of 5 March 2022, 10,704,043,684 doses of vaccines had been administered (https://covid19.who.int/; accessed on 12 March 2022). SARS-CoV-2 was discovered to be closely related to the genus Betacoronavirus [1], which is a member of the Coronaviridae family. SARS-CoV and MERS-CoV are some of the other members of this family [2]. This virus can spread through droplets and aerosols [3] with a very high infection and transmission rate [4].

SARS-CoV-2 has a positive-sense single-strand RNA (+ssRNA) as its genetic material [5]. RNA viruses have a high mutation rate, which is associated with increased virulence and adaptability, both of which are considered advantageous for viruses [6]. The mutations are primarily caused by errors in the viral RNA replication process, which results in the accumulation of sequences that undergo incorporation errors or go through recombination, giving rise to a variety of viral variants [7,8,9]. Certain variations in these genetic codes can weaken the virus, but can also increase its transmissibility, virulence, or ability to evade the body’s defense mechanisms [10]. The Omicron variant, or B.1.1.529 lineage, is one of the most recent variants to emerge as a result of the mutations that are currently spreading [11]. This variant has 37 amino acid changes in the spike (S) protein (compared to the delta variant), 15 of which are in the receptor-binding domain (RBD), which causes increased transmissibility [12].

A growing number of scientific communities are now questioning whether the Omicron variant may provide natural immunity as a result of its milder symptoms [13]. However, it is unknown whether the milder symptoms are caused by the virus acting as a natural immunization or by the fact that much of the human population has been vaccinated. The reason for this is that natural SARS-CoV-2 infection or vaccination results in the activation of complementary humoral (antibody) and cellular (T cell) immune responses [14]. Furthermore, despite the fact that it is spreading rapidly, this variant has a low hospitalization rate. Many people believe that this variant can act as a natural immunization and can train a variety of human immune systems. This review article discusses how the Omicron variant might or might not be able to provide natural immunity.

## 2. SARS-CoV-2 Mutation Results in the Emergence of Various Variants

Viruses evolve constantly as a result of mutation, and new viral variants are almost certain to emerge. Occasionally, new variants appear and then perish, while others remain in existence for a long time. During this pandemic, a large number of different SARS-CoV-2 variants have been monitored all over the world. Viruses with mutations in their genomes will dominate the population, regardless of their impact on viral fitness [7]. According to the Centers for Disease Control and Prevention (CDC), SARS-CoV-2 variants are classified into four classes, namely: “variants being monitored (VBM)”, “variants of interest (VOI)”, “variants of concern (VOC)”, and “variants of high consequence (VOHC)” (https://www.cdc.gov/coronavirus/2019-ncov/variants/variant-classifications.html; accessed on 31 January 2022) [15,16,17]. VBM are variants that have been linked to more severe illness or increased transmission, but are no longer detectable or are circulating at extremely low levels. These variants are no longer a threat to public health. As of 25 January 2022, variants Alpha, Beta, Gamma, Epsilon, Eta, Kappa, B.1.617.3, Zeta, and Mu were considered VBM [18].

**Figure 1:**
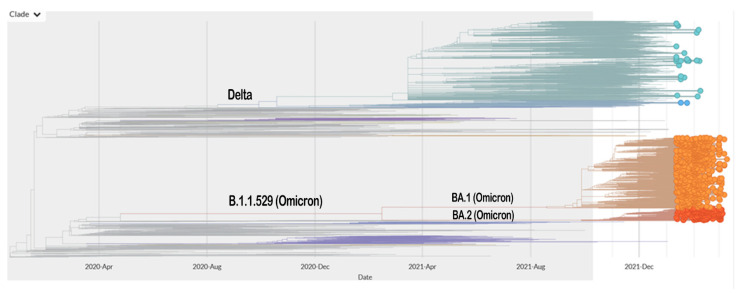
Phylogenetic tree of SARS-CoV-2 variants showing the position of the Delta and Omicron variants (https://nextstrain.org/ncov/gisaid/global?branchLabel=emerging_lineage&dmin=2022-01-12; accessed on 12 March 2022).

VOI are associated with altered receptor binding, decreased neutralization by antibodies produced in response to prior infection or vaccination, decreased treatment efficacy, diagnostic implications iota, or an anticipated increase in disease transmissibility or severity [19]. There is currently no variant in this category. VOCs are linked to higher transmissibility, more severe disease, a significant decrease in neutralization by antibodies developed from previous infections or vaccinations, lower therapeutic or vaccine efficacy, and diagnostic detection failures [19]. The Delta and Omicron variants were previously classified as VOCs. Figure 1 depicts a simplified phylogenetic tree of these significant variants.

Currently, three major lineages comprise the Omicron variant—BA.1, BA.2, and BA.3 [20,21]. Several studies have shown that Omicron BA.1 is able to escape neutralizing antibodies (NAbs) induced by vaccination or prior infection and, as a result, it has become increasingly prevalent in the population [22,23,24]. Nonetheless, Omicron BA.2 has recently been observed with increased frequency in several parts of the world, suggesting that this subvariant may have a selective advantage over BA.1 [25]. Hence, BA.2 possesses a strong chance of becoming the next predominant variant [26].

The BA.2 and BA.1 sub-variants share 12 of 16 amino acid substitutions in the receptor-binding domain (RBD) of the spike (S) protein. However, there are four amino acid substitutions (S371F, T376A, D405N, and R408S) in BA.2 RBD that differ from BA.1, suggesting that monoclonal antibodies against these different Omicron subvariants may perform differently [25,26]. BA.3 is only associated with a few cases of COVID-19, suggesting that this subvariant spreads at a very slow rate. This could be because six mutations (ins214EPE, S371L, G496S, T547K, N856K, and L981F) were lost from BA.1 or because two mutations were acquired from BA.2 (S371F and D405N) [21].

## 3. Concepts of Immunity

**Figure 2:**
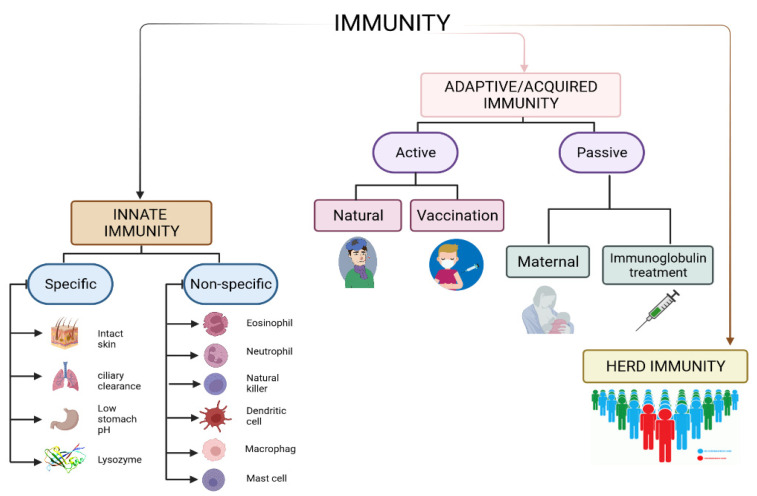
The types of immunity. Immunity refers to the ability of the body to prevent the invasion of foreign disease-causing microorganisms or substances. It is divided into innate and acquired immunity. The figure was prepared using Biorender (https://app.biorender.com; accessed on 14 March 2022).

Immunity is a physiological mechanism that enables the body to recognize foreign substances and neutralize, eliminate, or metabolize them without causing harm to its own tissues [27]. It is the state of being immune to a disease, which is achieved through the presence of antibodies as a proxy for immune protection against the particular disease in a person’s body [28]. Apart from physical and chemical barriers to pathogens, the immune system relies on two fundamental defense mechanisms: innate (nonspecific) immunity and adaptive (specific/acquired) immunity [29]. Some authors also incorporate herd immunity into this concept [30] (Figure 2).

### 3.1. Innate and Adaptive Immunity

Innate immunity is the first self-defense antigen-independent mechanism found in all multicellular organisms [31], which has the objective of preventing infection, eradicating invader pathogens [32], and activating the acquired immune response [29]. It is divided into specific and non-specific immunity. Specific-innate immunity includes physical barriers (such as the skin’s tight junctions), anatomical barriers (such as the mucous membrane), low stomach pH, and enzymes (such as lysozyme) [33]. On the other hand, nonspecific-innate immunity involves phagocytes (such as neutrophils, monocytes, and macrophages), and inflammation-related serum proteins (such as complement, CRP, and mannose-binding lectin) [31]. Due to the absence of immunologic memory in the innate immune response, it is incapable of recognizing or memorizing the same pathogen if the body is exposed to it again in the future [29].

Adaptive immunity refers to antigen-specific responses that are highly tailored to specific pathogens. It is tightly regulated by the interaction of innate immune cells [34]. Adaptive immune responses are the basis of an effective immunization process against infectious diseases [35]. The adaptive immune system is composed of two types of cells: antigen-specific T cells that are activated to proliferate by antigen presenting cells (APCs), and B cells that differentiate into plasma cells to produce antibodies. A group of proteins known as the major histocompatibility complex (MHC) is expressed on the surfaces of the APCs [29].

Adaptive immunity can be classified further into two types—active and passive immunity. Active immunity can be acquired naturally or as a result of vaccination. It develops as a result of a person’s direct infection with disease-causing organisms [36]. It occurs when a disease-causing organism stimulates the immune system to produce antibodies against that disease [29]. Antibodies are proteins that the body produces specifically to neutralize or destroy pathogenic organisms, their toxins, and fragments thereof [37]. Vaccine-induced immunity is acquired by vaccination with a killed or weakened form of the disease-causing organism [38,39].

In the case of active-acquired immunity induced by viral infection, for example, the immune system will respond immediately. At the time of infection, cytotoxic T-cells (also known as T_C_, cytotoxic T-lymphocyte, CTL, T-killer cell, cytolytic T-cell, CD8+ T-cell, or killer T-cell) will rapidly multiply to eliminate the infected cells [29]. They transport the virus to the lymph nodes. Afterwards, the major histocompatibility complex (MHC) class I-peptide complexes will be presented on the cell surface of an infected cell and activated CD8+ T cells. These T-cells are able to recognize the MHC class I-peptide complex and start to induce apoptosis of the infected cell by releasing cytotoxic granules [40]. Therefore, CD8+ T-cells are responsible for controlling viral infection [41]. Each of these cells has a T-cell receptor on its surface that recognizes specific antigens [42]. However, only a small proportion of T-cell receptors are antigen specific. These few antigen-specific T-cells divide rapidly and differentiate into effector T-cells in order to control the infection and maximize the defense against viruses. On the other hand, various types of helper T-cells will secrete chemical signals that stimulate other immune systems, such as B-cells. The killer T-cells eliminate the infected host cells prior to their demise once the infection is eradicated. Some of these short-lived effector cells differentiate into memory T-cells, which persist in the organism for an extended period of time [43]. If the same infection reintroduces itself into the body, memory T-cells are already present and prepared to fight the invader more effectively and quickly than during the initial contact [44]. Certain individuals may still have memory T cells from previous coronavirus infections, such as those that cause common colds, which are capable of recognizing SARS-CoV-2. These cells may aid in the fight against infection or may even completely eradicate it. It has been hypothesized that cross-reactive T cells prevent infection from taking hold [45].

Passive immunity, on the other hand, occurs when a person obtains antibodies from sources other than their own immune system [46]. For instance, a baby receives immunoglobulins from the mother, which are passed to her child through breastfeeding [47] or via the placenta [48]. This type of immunity can also be obtained through immunoglobulin therapy [49].

### 3.2. Herd Immunity

Herd immunity occurs when a sizable proportion of a population develops immunity to an infectious disease, thereby limiting the spread of the disease [50]. Then, it refers to the indirect protection conferred to susceptible individuals against infection. It can be achieved by vaccination programs and natural infection when there is a large enough proportion of immune individuals in a population [30]. If the proportion of the population immune to the disease exceeds a predetermined threshold, the disease’s spread will be reduced. This is referred to as the threshold of herd immunity. The herd immunity threshold is defined mathematically as 1 − 1/R_0_. R_0_ is the basic reproduction number, indicating the average number of secondary infections caused by a single infectious individual introduced into an entirely susceptible population [51]. The more transmissible a pathogen is, the greater its associated R_0_ and the greater the proportion of the population that must be immune in order to prevent sustained transmission [30]. If the estimated R_0_ value is greater than 1, the disease is still spreading [52]. According to the WHO, predicting how much of a population is immune and how long that immunity lasts will be difficult until COVID-19 immunity is better understood. For example, in order to achieve herd immunity against measles, approximately 95% of the population must be vaccinated in order for the remaining 5% to be protected, because measles will not spread among those who have been vaccinated. In the case of polio, the threshold is approximately 80–86% [53].

## 4. Vaccination

**Figure 3:**
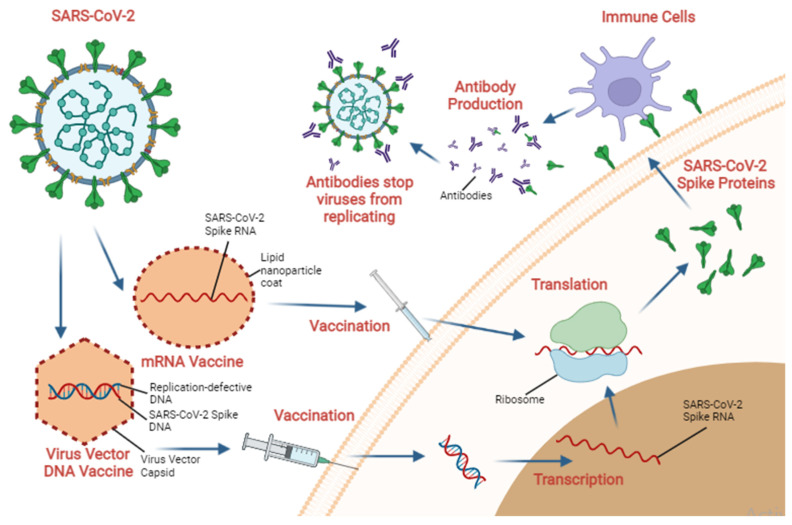
Schematic representation of how vaccination works. This image shows a simplified version of how the vaccine mechanism can block the SARS-CoV-2 virus from infecting a person’s body, starting with the manufacture of vaccines based on the SARS-CoV-2 virus. In this example, an mRNA vaccine contains the SARS-CoV-2 spike RNA. This RNA will be translated by the ribosome to make a spike protein, which can later be recognized by the immune system and produce antibodies that prevent the SARS-CoV-2 virus from replicating. There are also vaccines that use replication-defective viral vector DNA. This vaccine enters like a viral infection and then injects its DNA into the body. The body transcribes and translates it, but the spike protein is only produced later. The figure was prepared using Biorender (https://app.biorender.com; accessed on 01 February 2022).

Immunization is the process by which the body recognizes foreign substances and gains immunity against a disease through vaccination [54]. Meanwhile, vaccination is the process of administering a vaccine to assist the immune system in developing immunity against a disease [55]. Numerous vaccines have been developed to prevent infection and the spread of SARS-CoV-2. Vaccines are biological preparations that are intended to safely induce an immune response, thereby protecting against infection by microorganisms (e.g., viruses) [38]. In order to build an immune response, vaccines must consist of components of the pathogen that act as antigens, whether derived from the pathogen itself or synthetically produced to represent components of the pathogen [38]. These include live attenuated viruses (viruses that have been weakened or altered in such a way that they no longer cause illness), inactivated or killed organisms or viruses, inactivated toxins (for bacterial diseases), or only segments of the pathogen (this includes both subunit and conjugate vaccines). The body stores information about these recognized antigens as a memory that will be used to fight these pathogens in the future [56]. Figure 3 illustrates the vaccine’s mechanism of action schematically.

Several vaccines have been approved, and a global mass vaccination campaign is currently underway. Vaccination continues to be a critical component of the multi-layered approach required to mitigate the impact of Omicron, while also addressing Delta’s ongoing circulation, as stated by the European Centre for Disease Prevention and Control (ECDC) (https://www.ecdc.europa.eu/sites/default/files/documents/covid-19-assessment-further-emergence-omicron-18th-risk-assessment-december-2021.pdf; accessed on 31 January 2022). In comparison to other previously available variants, there is compelling evidence that preventive or medical measures have significantly reduced VOHC’s effectiveness. According to the CDC, there is currently no variant in this category that is circulating worldwide. On the other hand, the World Health Organization (WHO) has a different approach to the classification of SARS-CoV-2 variants. WHO categorized the variants as VOI, VOC, and variants under monitoring (VUM) (https://www.who.int/en/activities/tracking-SARS-CoV-2-variants/; accessed on 31 January 2022).

## 5. Omicron Variant and Its Characteristics

VOCs are variants that meet the VOI criteria and also exhibit characteristics such as increased transmission rates or alarming changes in COVID-19 epidemiology, as determined through careful assessment and research [57]. Additionally, they increase the likelihood of contracting COVID-19, enhance the virus’s ability to infect, and impair the efficacy of diagnostic tools, vaccines, and treatments. The Alpha, Beta, Gamma, Delta, and Omicron variants were included in this category (Table 1).

### 5.1. Omicron Variant and Its Characteristics

The Omicron variant was designated as a VOC by the World Health Organization on 21 November 2021. This variant was discovered for the first time in Botswana (Southern Africa) on 11 November 2021. Since then, it has spread throughout the world. On 12 November 2022, this variant became dominant in South Africa’s province of Gauteng, where 77 samples were identified as Omicron variants. It spread very quickly across the country, with the average number of COVID-19 cases rising from 280 to 800 per day after the Omicron variant was confirmed [11].

Phylogenetic analysis indicates that the Omicron variant diverged early from other SARS-CoV-2 variants, rather than evolving from the preceding VOCs. The sequence analysis revealed a significant difference between the Omicron variant and the other variants, making it difficult to determine which variants it originated from [64]. There is a hypothesis that this variant originated in immunocompromised individuals or that it did not even originate in humans [65].

**Figure 4:**
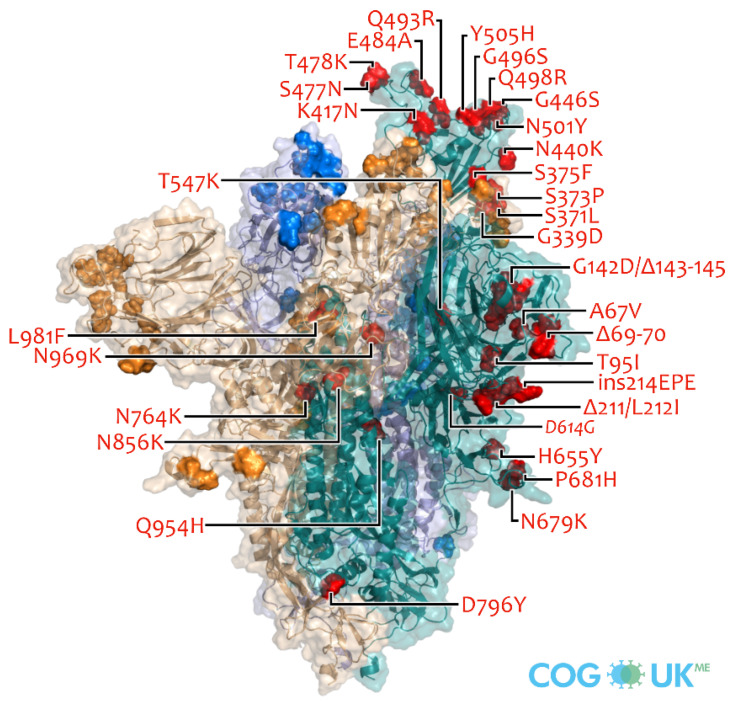
Omicron variant spike protein mutations (MRC-University of Glasgow Centre for Virus Research) (https://sars2.cvr.gla.ac.uk/cog-uk/mutants_BA.1_ME_web.png; accessed on 31 January 2022).

The Omicron variant is unique in that it contains the most mutations of all SARS-CoV-2 variants (Figure 4). A total of 50 mutations have occurred in this variant [66]. The envelope (E) protein has one substitution (T9I), the membrane (M) protein has three substitutions (D3G, Q19E, and A63T), and the nucleocapsid (N) protein has three substitutions and a three-residue deletion. The Omicron variant has six amino acid changes (K856R, L2084I, A2710T, T3255I, P3395H, and I3758V) and two deletions (amino acid 2083 and amino acids 3674–3676) within ORF1a. The variant comprises two changes within ORF1b (P314L and I1566V). ORF9b also contains a P10S change and a three-residue deletion at positions 27–29 (https://covariants.org/variants/21K.Omicron; accessed on 25 January 2022). Compared with the early variant of SARS-CoV-2 (Wuhan-hu-1), Omicron has more than 30 mutations in the spike protein (substitutions of L981F, N440K, T95I, Y145D, L212I, G339D, S371L, S373P, S375F, K417N, G446S, S477N, T478K, E484A, Q493R, G496S, P681H, Q498R, N501Y, Y505H, T547K, D614G, Q954H, H655Y, N679K, N764K, D796Y, N856K, N969K, and A67V; three deletions of N211, G142/V143/Y144, and H69/V70; and one insertion of three amino acids at position 214) [67]. These mutations occur in both RBD and NTD, which is the primary target of neutralizing antibodies, such as mAbs and nanobodies (Nbs) [68].

A study found that 20 mutations in the poly-mutant spike protein (PMS20) are sufficient to prevent polyclonal neutralizing antibodies from individuals exposed to SARS-CoV-2 or those who have received the vaccine twice [69]. Mutations in the Omicron variant are similar to or even more severe than the PMS20 mutation. The early doubling times of the Beta, Delta, and Omicron variants were calculated to be 1.7, 1.5, and 1.2 days, respectively. This finding suggests that the Omicron variant is more infectious than the Delta and Beta variants. As a result, it is more contagious [11].

The Omicron variant excels at evading neutralizing antibodies. Cao et al. [70] demonstrated that 85% of 247 human RBD-targeted neutralizing antibodies (NAbs) were escaped by Omicron. There was an indication that Omicron would result in significant humoral immune evasion. On the other hand, NAbs targeting the conserved Sarbecovirus region remained the most effective. In the plasma specimens from people who had recovered from COVID-19, 50% neutralization titer (NT_50_) values were 32–58% lower for omicron compared to Wuhan-hu-1. Additionally, NT_50_ values for omicron were 127 times lower than those who had received two doses of an mRNA vaccination 1.3 months before sampling than for Wuhan-hu-1. The neutralizing potency of Omicron was 27 times lower 5 months after vaccination [69].

It was suggested that a third vaccine dose was necessary because the Omicron variant had a significantly higher neutralization efficiency (by a factor of 100) after the third dose than after the second dose. Despite this, neutralization against the Omicron variant was significantly lower (by a factor of four) than that against the Delta variant, even after three vaccine doses [71]. However, a significant Omicron neutralizing activity was observed in individuals who had recovered from COVID-19, or after a third dose of mRNA vaccine was administered to individuals who had received at least 6 months after the second dose of an mRNA vaccine [69].

Despite a high infection rate, the Omicron variant has milder symptoms and lower hospitalization rates compared with individuals infected with previous variants [63]. Additionally, Omicron infection was reported to be associated with a significantly lower risk of severe clinical outcomes and a shorter length of stay in a Southern California hospital [72]. Another study reported that the hospitalization rate of Omicron was lower than for the Beta and Delta variants in South Africa, and it was also reported to show milder symptoms [73]. As a result, some experts believe that Omicron is a good sign [74].

The exact reason for Omicron’s lower hospitalization and mortality rates is still under research. One possible explanation is that the mutations in Omicron enable it to infect the upper respiratory system more effectively than the lower respiratory system [75]. As a result, the infected person’s lung remains relatively unaffected. The lower hospitalization rate of Omicron is supported by the fact that Omicron has a higher viral load, which means that the amount of virus shed is greater than that of Delta [76]. However, because the upper respiratory system is more susceptible to infection from the outside world, this is likely another reason it is so contagious. This is reinforced by a study that discovered that the majority of people infected with Omicron are asymptomatic, allowing them to spread the infection without even realizing it [77].

While Omicron is less severe, it remains a threat, particularly to those with immunocompromised conditions and the elderly [78]. As Omicron spreads, it will increase the chance of infecting vulnerable people. While it has a low hospitalization rate in percentage terms due to its infectious nature, it can still place a strain on healthcare systems.

### 5.2. The Rationale for Presuming That Omicron Confers Natural Immunity

It has previously been observed that a virus that causes a pandemic that continues to mutate eventually becomes less severe. The Bubonic plague, also known as the Black Death, killed more than a third of Europe’s population (approximately 25 million people), during the 14th century. The disease was caused by *Yersinia pestis*, a bacterium that causes plague in humans and other mammals [79]. The exact cause of the pandemic’s demise is still unknown. However, several experts have proposed hypotheses, one of which is a combination of quarantine and bacterial genetic changes. It was presumed that the bacteria responsible for this pandemic mutated. This is demonstrated by the disease’s recurrence every century, but its impact is diminishing. It was believed that this disease appeared for the last time in the 18th century, but it was not nearly as deadly as the first appearance [80]. Perhaps the bacteria evolved into a natural form of vaccination, eventually rendering the remaining population immune, or perhaps the bacteria evolved into a less lethal strain. We speculate that this scenario is quite likely to occur with Omicron, although extreme caution should be exercised.

The Spanish flu appears to have followed the same pattern. At the time, there was no specific vaccination or drug available to bring the pandemic to an end. The pandemic was believed to have ended or gradually waned in severity as a result of survivors’ developing immunity (herd immunity) and the virus mutating. This flu also reappeared a few years later, but was far less lethal than the first time. According to some reports, a similar phenomenon will occur with COVID-19 today [81,82].

As previously described, the Omicron variant exhibits greater transmissibility than the previous variants, but also exhibits milder symptoms and a lower rate of hospitalization [83,84]. Due to these facts, many scientists now argue that this variant provides natural vaccination because it can infect a large number of people while causing less harm, allowing it to serve as a natural vaccination that trains the immune systems of a large number of people. Additionally, some argue that obtaining immunity from the Omicron variant is preferable because it contains the most recent mutation (the most current) in comparison to several artificial vaccines that use the older variant base [85]. One particular study discovered that people who received the BioNTech-Pfizer vaccination but had never tested positive for COVID were 13 times more likely to contract the Delta form than those who had been infected but had not been vaccinated. Despite the fact that it has not yet been peer reviewed, that study is now the largest comparison of immunity gained from previous COVID-19 infection to vaccine-induced immunity, with over 30,000 patients having participated in the survey [86].

The best defense comes from a combination of different sources of immunity. Additionally, individuals who receive multiple types of vaccines have stronger immune systems [87]. According to one study, the best outcomes were seen in people who had a combination of immunity from prior infection and two vaccinations. Immunologists refer to this phenomenon as “hybrid” and “super” immunity [88]. Super immunity is expected to ward off future waves [89].

There is a widespread belief that Omicron infection will provide long-term immunity. The fact is that when someone has a high virus load, such as in the case of being infected by Omicron, it indicates that they have been severely infected by the virus [76]. This enables Omicron to easily penetrate the body’s cells, eliciting a stronger immune response. According to some studies, Omicron stimulates the immune system more than Delta [90], implying that it will provide longer immunity. It is unknown, however, whether it confers lifelong immunity. Infection-induced immunity and vaccination-induced immunity have been shown to have comparable rates. The Pfizer-BioNTech or Oxford-AstraZeneca two-dose vaccinations, in particular, provide comparable protection to infection-induced immunity. During the Alpha variant wave, two doses of the vaccine provided 79% protection, while infection-induced immunity provided 65% protection. However, during the Delta variant wave, two doses of the vaccine provided 67% protection, while infection-induced immunity provided 71% protection [91].

**Figure 5:**
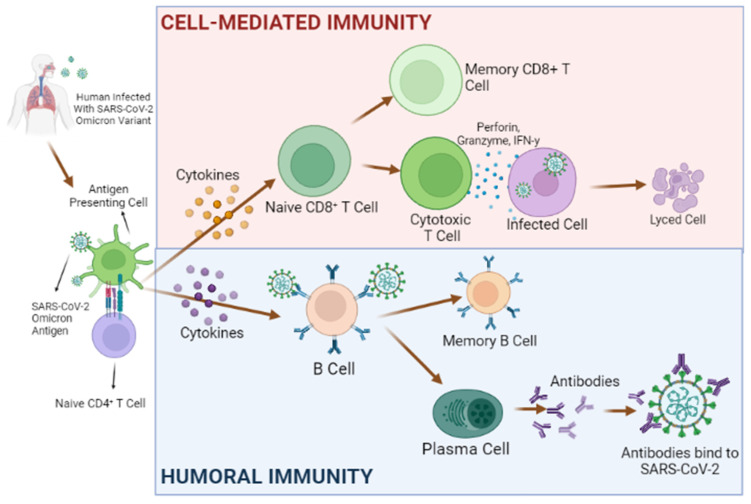
Omicron infection as a natural way of vaccination works by triggering infection-induced immunity, incorporating both humoral and cell-mediated immunity. Adapted from Schijns et al. [102]. The figure is created using Biorender (https://app.biorender.com; accessed on 1 March 2022).

Infection-induced immunity from Omicron variants with milder symptoms may elicit fewer antibodies compared to the severe variant infection immunity [92,93], but it is probably more long-lasting than most vaccines [94,95,96,97]. The reason is that most vaccination-induced immunity is based on serum antibody titers and efficacy measurements are also based on antibodies [98,99], while infection-induced immunity has additional T-cell-mediated immune responses [100,101] (Figure 5). Johnson and Johnson is the only vaccine provider that has publicly stated that their vaccine provides both mechanisms (https://www.jnj.com/johnson-johnson-single-shot-covid-19-vaccine-demonstrated-a-durable-immune-response-and-elicited-dual-mechanisms-of-protection-against-delta-and-other-sars-cov-2-variants-of-concern-in-data-published-in-new-england-journal-of-medicine; accessed on 3 February 2022).

Antibodies can only detect proteins outside of the cells, and several coronavirus vaccines target the spike protein that adorns the virus’s surface [103]. However, because the spike protein is prone to mutation, new forms will be able to elude antibody detection [104]. On the other hand, T-cells can target viral proteins that are produced inside infected cells, some of which are quite persistent (https://covariants.org/variants/21K.Omicron; accessed on 3 February 2022). Because T-cells are only activated once a virus has penetrated the body, they cannot prevent infection. However, they are critical in the treatment of infections that have already begun by destroying virus-infected cells [40].

T-cells have been shown to fight COVID-19 in the long term and are more resistant to new variants [94,95,96,97]. A study demonstrated that 17 years after infection with SARS-CoV during the 2003 pandemic, 23 patients who had recovered from SARS syndrome still had CD4 and CD8 T-cells. Despite the subjects’ claims that they had never had COVID-19, some of those cells showed cross-reactivity against SARS-CoV-2 [105].

In our opinion, whether or not the Omicron variant serves as a natural vaccination, the disease that caused the pandemic typically weakens and eventually disappears over time. According to one study, SARS-CoV-2 was predicted to become a seasonal virus with low mortality [106]. First, the mortality and severity of SARS-CoV-2 infection were age and comorbidity dependent, and second, infection-induced immunity is long-lasting and excellent when combined with vaccination. When younger individuals contract this virus, it is assumed that they will develop immunity from an early age, similar to other children’s vaccination programs. The Omicron variant has been shown to be more prevalent in younger individuals and to have milder symptoms [107].

We presume that this notion leads people to believe that the disease will become less contagious over time as the causal agent continues to mutate. The majority of mutations are detrimental to the organisms that carry them. Because many mutations result in organisms leaving fewer descendants over time, natural selection acts to eliminate these mutations from the population [6]. In other words, having an excessive number of mutations becomes a burden, eventually resulting in their eventual extinction. The “avirulence theory” and “Theobald Smith’s Law of Declining Virulence” are two terms used to describe this [108]. It appears that disease-causing hosts benefit from mutating to be less virulent.

In accordance with evolutionary survival of the fittest principles, when a virus quickly kills its host, it does not have as much time to spread. On the other hand, a virus that kills its host slowly or does not result in death has more time to spread. This is called the trade-off model, which has been previously described [109]. We speculate that a consequence of this is that more severe viruses become less prevalent in the population, whereas less severe viruses become increasingly prevalent in the population. This means that the virus must undergo mutations in order for natural selection to be effective.

It should be noted that this thought or conjecture is not entirely correct. In the cases of several other diseases, such as rabies [110] or tuberculosis [111], there has been no evidence of a reduction in the severity of the effects of infection. It is possible for them to survive even after eliminating 100% of the human population, by mutating and infecting other animals to serve as their hosts [112].

RNA viruses have a higher rate of mutation than DNA viruses. Mutation rates range from 10^−8^ to10^−6^ s/n/c for DNA viruses and from 10^−6^ to 10^−4^ s/n/c for RNA viruses [113]. Likewise, single-stranded viruses mutate more quickly than double-stranded viruses do [114]. Virus mutations are essentially unpredictable. However, a recent study discovered that mutations might not be a random event [115]. The mutation could be caused by viral replication errors within the host cell in the absence of a repair mechanism. The mutation rate of viruses is defined as the rate at which errors are introduced into the viral genome during replication. Because the vast majority of mutations are harmful, mutators will suffer from an increased mutation load and will be at a disadvantage in comparison to competitors who have a lower mutation rate [9].

A mutation may be meaningless [116], or it may reduce the symptoms of infection [117], or it may cause the mutator to become more virulent [118]. However, in our opinion, although there is no guarantee that the next variant after Omicron will be less virulent, and this trend will likely continue until COVID-19 is classified as a common cold. Contracting an Omicron variant voluntarily is something to avoid, as the virus could evolve into a more lethal variant. Because the virus mutates, resulting in changes (whether beneficial or detrimental), humans must also adapt. Parvovirus B19V and hepatitis B viruses can be used as examples of how their prolonged presence in a host can result in a change in their genotype [119]. We assume that this coevolutionary process may eventually benefit humans by increasing immunity through vaccines and limiting the spread of Omicron. Perhaps the simplest way for viruses to start a new epidemic is to gradually evade immunity. This is consistent with previous observations regarding seasonal coronaviruses. A new epidemic model that takes into account multiple viral strains as well as reinfection due to waning immunity has been proposed [120].

**Figure 6:**
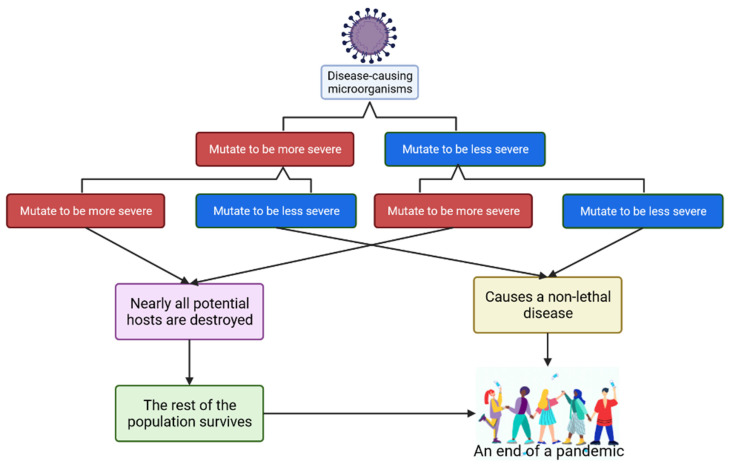
A hypothesis that describes how a pandemic end due to a series of mutations.

**Figure 7:**
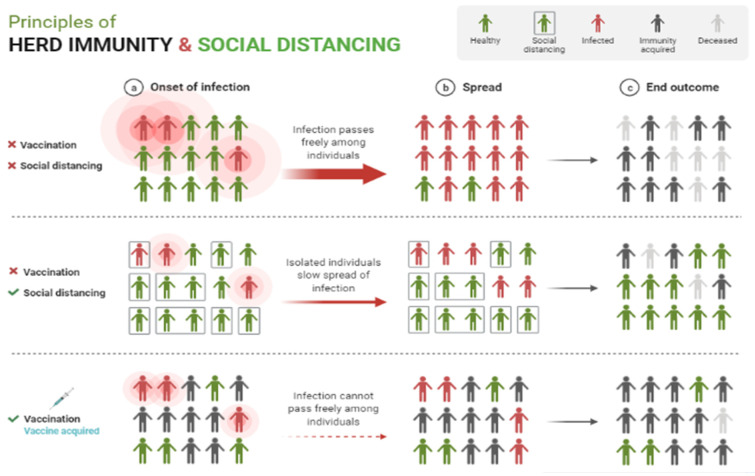
How a pandemic end by incorporating vaccination and social distancing to achieve herd immunity. Adapted from “Principles of Herd Immunity and Social Distancing” (source: https://app.biorender.com; accessed on 3 February 2022).

The emergence of the Omicron variant may be fortunate, because it results from a mutation that increases the viral transmissibility and infectivity, but results in milder symptoms. Our interpretation based on the current phenomenon is presented in Figure 6. We considered that previous pandemics may have been the result of a series of fortunate events that allowed the virus to continue to mutate and become less fatal. Thus, the disease became less lethal, transforming it into a common disease with a low mortality rate and effectively ending the pandemic. Lethal mutations cause their hosts to die more quickly, reducing their chances of spreading and allowing them to be isolated successfully. For example, if the Delta variant was extremely lethal, it would eventually eliminate all individuals who contracted the virus, thus preventing the virus from spreading. This would be a controversial approach, but it would put an end to the pandemic. This approach could work side by side with herd immunity, which is a common theory of eradicating pandemics. Although the threshold has not been defined, 60–80% immunity from the entire population is typically required. At this rate, the virus would start to diminish over time. This starts with the introduction of a novel pathogen, then leads to most of the population getting infected and then developing immunity to it, and then ends when many people are immune to it. This process can produce safer results and can be accelerated with the incorporation of vaccination and social distancing (Figure 7).

There is still a possibility that some viruses will become more virulent, prolonging the process of eradicating the pandemic. The only way to end this pandemic is to keep Omicron as the last variant. This can be accomplished through vaccination and the avoidance of infection-causing agents, namely SARS-CoV-2. However, keeping Omicron as the last variant might be a great challenge. The Omicron variant originated in South Africa, and only about 9% of the population has been vaccinated across the entire African continent [121]. The emergence of new variants could be aided by the large number of unvaccinated people and widespread SARS-CoV-2 replication, thus raising concerns. Today, our world is divided into countries that have implemented the fourth dose [122], while others still have the majority of their population unvaccinated. There is currently insufficient scientific evidence to support the importance of fourth doses [91].

Natural immunity from other SARS-CoV-2 variant infections may provide as little as 19% protection against Omicron [63]. Thus, in order to maintain the momentum of new variants with progressively milder symptoms, it is prudent to continue vaccinations and not to rely solely on immunity from the Omicron variant to combat the next variants. As a result, it is hoped that the subsequent variants will remain milder, and that this will be a process leading to the end of the COVID-19 pandemic, similar to previous pandemics.

While there is considerable scientific speculation that Omicron may act as a natural vaccine, the CDC believes that this cannot be used as a guide and that health protocols must be followed and the vaccination program must be completed. As a result, rather than speculating about the possibility of intentionally becoming infected with Omicron in order to gain immunity from it, research findings should be taken into consideration.

## 6. Conclusions

After considering all of the evidence, we believe it is highly likely that the Omicron variant confers natural immunity and that this is an early stage of what has happened in previous pandemics. SARS-CoV-2 is assumed to be overburdened with mutations and that it will eventually become less lethal over time. The Omicron variant may not be the final variant, but rather the first in a series of processes that will eventually bring the COVID-19 pandemic to an end, or transform it into a common disease with few or no fatalities. The prudent course of action is to adhere to the vaccination schedule and health protocols recommended thus far. However, there is still a risk of severe symptoms as a result of the Omicron variant, particularly in those at high risk and those with comorbid conditions. Compliance with health protocols and booster vaccinations will be significant factors in ending the COVID-19 pandemic process more quickly in comparison to previous pandemics that lasted decades or even centuries. These pandemics lacked modern information systems, detailed medical knowledge, and the best treatment available at the time, and perhaps could have been averted. We hope that the SARS-CoV-2 Omicron variant is the most recent one against which the community has developed immunity, or that new variants with diminishing symptoms continue to emerge, allowing this pandemic to evolve into a new endemic.

## Figures and Tables

**Table 1:** SARS-CoV-2 variants of concern.

Greek Name	First Identified	Noteworthy Mutations	Transmission	Hospitalisation
Alpha	20 September 2020, England	N501Y, P681H	+29% [58]	+52% [59]
Beta	May 2020, South Africa	K417N, E484K, N501Y	+25% [58]	Under research
Gamma	November 2020, Brazil	K417T, E484K, N501Y	+38% [58]	Increased [60]
Delta	October 2020, India	L452R, T478K, P681R	+97% [58]	+85% [61]
Omicron	9 November 2021, South Africa	P681H, N440K, N501Y, S477N, and numerous others	Increased [62]	−41% [63]

## Data Availability

Not applicable.
